# Early Childhood Oral Health Impact Scale (ECOHIS) questionnaire: reliability generalization meta-analysis of Cronbach’s alpha

**DOI:** 10.1186/s12903-025-06342-2

**Published:** 2025-06-09

**Authors:** Kalyana C. Pentapati, Deepika Chenna, Vijay S. Kumar, Nanditha Kumar, Mithun Pai, Saurabh Kumar

**Affiliations:** 1 Department of Public Health Dentistry, Manipal College of Dental Sciences, Manipal, Manipal Academy of Higher Education, Manipal, Karnataka 576104 India; 2 Department of Immunohematology and Blood Transfusion, Kasturba Medical College, Manipal, Manipal Academy of Higher Education, Manipal, Karnataka 576104 India; 3 Department of Public Health Dentistry, Amrita School of Dentistry, Amrita Vishwa Vidhyapeetham, Kochi, Kerala India; 4 Department of Prosthodontics, JSS Dental College & Hospital, JSS Academy of Higher Education & Research, Mysuru, Karnataka India; 5 Department of Public Health Dentistry, Manipal College of Dental Sciences, Mangalore, Manipal Academy of Higher Education, Manipal, Karnataka 576104 India; 6 Department of Pedodontics and Preventive Dentistry, Manipal College of Dental Sciences, Manipal, Manipal Academy of Higher Education, Manipal, Karnataka 576104 India

**Keywords:** Children, Internal consistency, Oral health, Quality of life, Questionnaire

## Abstract

**Objective:**

To summarise the estimates of Cronbach’s α for the Child Impact Section (CIS), Family Impact Section (FIS), and total Early Childhood Oral Health Impact Scale (ECOHIS) and to identify the significant predictors that may influence the pooled estimates.

**Materials and methods:**

A systematic search was conducted in PubMed, Scopus, EMBASE, Web of Sciences, Dentistry and Oral Science Source, and CINAHL from inception until 21st November 2024. Included were those reported in English with Cronbach’s α values for the ECOHIS or its subscales. Abstracts, conference proceedings, letters to editors, pilot studies, brief communications, and commentary were omitted. Two review authors independently reviewed the title and abstracts and full-text. Data extraction included demographic characteristics, language, study design and setting, sampling, Cronbach’s α, and questionnaire item count. COSMIN checklist was used for quality assessment and “Reliability Generalization Meta-analysis” (RGMA) was conducted to estimate the internal consistency of ECOHIS questionnaire.

**Results:**

Overall, 1,524 publications were retrieved, of which 454 and 304 were available for screening title and abstracts and full texts respectively. Among the included 66 publications, 74 estimates were obtained. The pooled Cronbach’s α for the total scale was 0.86 with high heterogeneity (I^2^ = 95.43). No significant impact of moderators was seen on the pooled estimate. Subgroup analysis showed little variation in the pooled estimates for continent, language, study setting, study design, and type of sampling. Only 46 estimates were included for the RGMA of CIS (0.85) and FIS (0.79). No significant effects of moderators were seen on the pooled Cronbach’s α for both CIS and FIS. Similarly, subgroup analysis showed little variation in the pooled estimates for the CIS and FIS for various categorical predictors.

**Conclusion:**

The pooled Cronbach’s α of ECOHIS and its sub-scales were higher than the acceptable benchmark with no effect of moderators.

**Supplementary Information:**

The online version contains supplementary material available at 10.1186/s12903-025-06342-2.

## Introduction

“Oral health-related quality of life” (OHRQoL) is individual’s perception of their oral condition and its effect on their daily activities, such as eating, smiling, sleeping, psychological status, and interaction with others [1]. Oral conditions can affect an individual’s life, even among children. Hence, assessment of OHRQoL helps to focus on the individual’s social, emotional, and physical functioning. Many questionnaires exist to evaluate the OHRQoL, while only a few instruments like the “Child perception questionnaire” and “child– Oral Impacts on Daily Performances” (C-OIDP) are available for children and adolescents [2]. “Early Childhood Oral Health Impact Scale” (ECOHIS) and “Scale of Oral Health Outcomes (SOHO)” -5 have been developed to capture the OHRQoL among young children. ECOHIS is a parent-reported questionnaire developed by Pahel et al. [3] with 13 items rated on a 5-point likert scale “never”, “hardly ever”, “occasionally”, “often” and “very often”. Involving parents in the evaluation of OHRQoL is crucial because preschool-aged children are cognitively underdeveloped and may not be able to contextualize and report the effects produced by oral problems. The questionnaire has “Child Impact Section” (CIS) (9 items divided into “child symptom”, “child function”, “child psychology”, and “child self-image” and “social interaction” sub-sections) and “Family Impact Section” (FIS) (4-items divided into “parental distress” and “family function” sub-sections).

ECOHIS has been widely used to assess OHRQoL in diverse clinical conditions. They include caries [4–9], gingival bleeding [10, 11], bruxism [12], cleft lip and palate [13], and oral hygiene [14]. Additionally, it was applied in studies that involved traumatic dental injuries [15–18], malocclusion [14, 15, 17], developmental dental defects [19], dental attendance [20], toothache [21] and orofacial pain [22] demonstrating its wide range of applications. It was validated with a variety of measures that include global oral health [23, 24], global ratings [11, 25–28], self-reported oral health [29], level of satisfaction [20], oral health literacy [30], oral health behavior [19], perceived oral health [19], and dental procedures performed in general anesthesia [31]. It was developed in English and translated to Arabic [24, 32], Brazilian [12, 16, 21, 33–41], Brazilian Portuguese [5, 8, 13, 42, 43], Chinese [31, 44, 45], Croatian [46], Farsi [20], French [47], German [48], Greek [49], Hindi [50], Indonesian [25, 51], Kiswahili, Luganda [19, 52], Lithuanian [26, 28, 53], Lumasaba [54], Malagasy [29], Malay [9, 55], Malayalam [23], Nigerian Pidgin English [56], Norwegian [22, 57], Sinhalese [10], Slovenian [58], Spanish [6, 14, 15, 17, 59–61], Swedish [62], Taiwanese Mandarin [63], Tamil [64], Thai [65], and Turkish [11, 18, 66] languages. The Cronbach’s α for the total ECOHIS questionnaire ranged from 0.519 to 0.97, higher than the recommended cut-offs (0.7) [67] for most of the studies. The alpha values for the CIS ranged from 0.707 to 0.95 and FIS ranged from 0.46 to 0.96. It has been administered to parents of high-risk children like intellectually disabled [42], oral clefts [13], autism [33, 34], HIV [52, 68], children with special health care needs [39], and juvenile idiopathic arthritis [22, 57].

Reliability Generalization Meta-Analyses (RGMA) were reported on different OHRQoL questionnaires [69–72]. Nevertheless, there was no information available on the RGMA of the ECOHIS questionnaire. This RGMA helps to understand the average and variability of reliability coefficients of ECOHIS questionnaire and to identify the potential variables that may have influenced estimates. With this background, it was aimed to summarize the Cronbach’s α for ECOHIS and its subscales. The objective was to perform RGMA of the coefficients of α for ECOHIS and its subscales (CIS and FIS) and to identify potential predictors that may have influence on the pooled estimates of these scales.

## Materials and methods

This RGMA was drafted as per “Reporting Quality of Reliability Generalization Meta-Analyses” (REGEMA) guidelines and the protocol was registered with PROSPERO on 2nd December 2024(CRD42024616535).

### Search strategy

A systematic search was performed in “PubMed”, “Scopus”, “EMBASE”, “CINAHL”, “Web of Science”, and “Dentistry and Oral Science Source” from inception until 21^st^ November 2024. The search terms used were “Early Childhood Oral Health Impact scale OR ECOHIS” as per the previous RGMA on OHRQoL questionnaires [69–72].

### Criteria for the inclusion and exclusion of publications

Included publication were those in English-language with Cronbach’s α values for the ECOHIS questionnaire. Abstracts, conference proceedings, letters to editors, pilot studies, brief communications, and commentary were omitted.

### Screening and extraction of data from publications

Screening and duplicate removal were performed using “Rayyan” (https://www.rayyan.ai/). The abstracts and titles were independently reviewed in duplicate (KCP and VK) followed by the full-text screening of the eligible studies in duplicate. The third reviewer (CD) resolved the disagreements. The variables extracted were based on the previous RGMA [69–72]. All the reviewers were trained and calibrated and used standardized form for data extraction. They were author name and year, continent, sample size, age and sex distribution, study setting, design, sampling methods, Cronbach’s α for CIS, FIS, and total scale, number of items, and language of administration.

All the studies were subjected to quality assessment with the help of “COSMIN checklist for internal consistency (Box 4)” [73]. The checklist had 3-items viz., “Was an internal consistency statistic calculated for each unidimensional scale or subscale separately?”, “For continuous scores: Was Cronbach’s α or omega calculated?” and “Were there any other important flaws in the design or statistical methods of the study?”. Each item was graded as “very good” or “adequate” or “doubtful” or “inadequate”. An overall grade was determined by considering the lower score of the above 3-items.

### Statistical analysis

All the analyses were conducted in “Jamovi software” (Version 2.6.23; MAJOR module version 1.2.4 https://www.jamovi.org) [74]. Heterogeneity was estimated using I^2^ and Q statistics. Due to the high heterogeneity, “random effects model” was used for RGMA (“restricted maximum likelihood method”). Subgroup analysis was performed for categorical variables like continent, language, study setting, design and sampling. “Publication bias” was estimated with “Egger’s regression coefficient” and the “funnel plot”. Meta-regression was used for continuous variables to understand the impact of study level characteristics on the Cronbach’s α. RGMA, subgroup analysis, and meta-regression were performed for ECOHIS and its subscales independently. Studies that lacked information on various predictors were excluded in meta-regression.

## Results

**Fig. 1:**
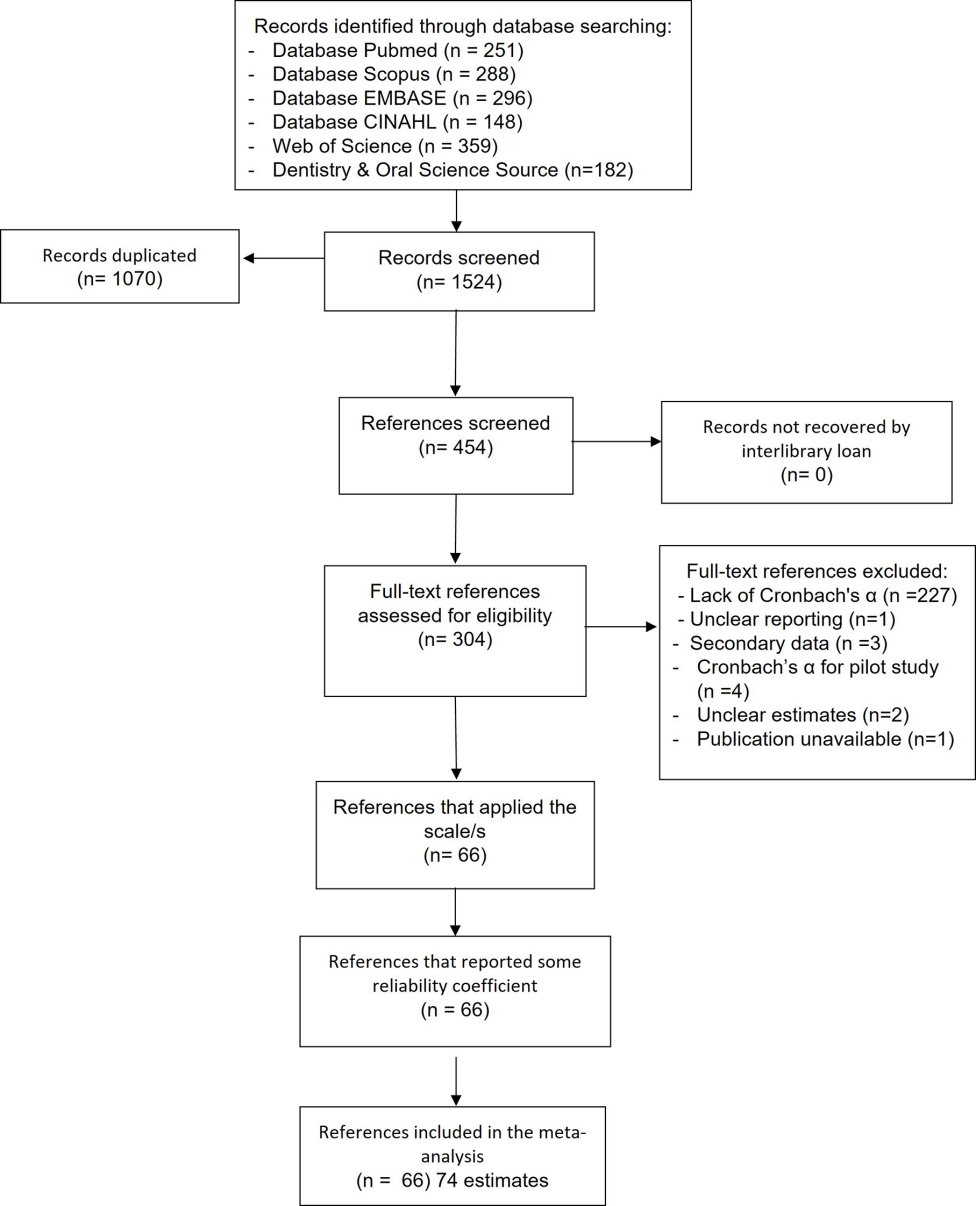
REGEMA flowchart

A total of 1,524 publications were retrieved. After screening titles and abstracts, 454 were selected, and 304 were assessed at full text level. Seven publications did not report the Cronbach’s α for the ECOHIS questionnaire [3, 10, 17, 19, 27, 35, 44] and six publications have yielded more than one estimate [19, 22, 31, 42, 57, 60]. Only 46 publications reported Cronbach’s α for CIS and FIS scales (Fig. 1). Two publications reported Cronbach’s α only for CIS [19] and FIS [17] scales respectively.

The Cronbach’s α for the total ECOHIS scale ranged from 0.519 to 0.97 (mean: 0.86; SD: 0.07; Median: 0.85). The Cronbach’s α was higher than the recommended (0.7) [75] for almost all the studies except for one study [51]. The alpha values for the CIS ranged from 0.71 to 0.95 and were above the recommended cut-off. However, the alpha values for the FIS ranged from 0.46 to 0.96 and nine studies reported estimates less than the recommended cut-off [5, 13, 27, 28, 35, 43, 46, 57, 62].

### Age distribution of the children

The mean age was not reported in 21 estimates [5, 6, 9, 14, 17, 19, 25, 29, 32, 40, 44, 47, 52, 57, 59, 61, 63, 64, 66] and eight estimates have not reported the age ranges [4, 21, 27, 35, 59, 76, 77]. One study have not reported age range or median or mean [59](Table 1).

### Sex distribution of the children

Ten estimates did not report the sex distribution [3, 13, 30, 42, 49, 51, 57, 59]. This analysis included 9954 boys and 9412 girls (Table 1).

### Study setting and design

Most of the included publications were cross-sectional except for 16 estimates [7, 22, 28, 31, 34, 37, 40, 52, 54, 60, 76, 77]. Most of the estimates (*n* = 35) were from school / community settings [3, 6, 8–11, 15, 17, 19, 23, 25, 29, 30, 32, 35, 36, 38, 41–44, 50, 51, 54, 55, 58, 59, 61, 63, 65, 76–78] with a pooled alpha of 0.85, 0.82 and 0.9 for CIS, FIS and total ECOHIS respectively (Table 2). There were minimal differences in the pooled estimates across study designs for CIS, FIS and total scales.

### Continent

Majority of the estimates of ECOHIS questionnaire were conducted in South America (*n* = 21), Asia (*n* = 19), and Europe (*n* = 14). The cumulative alpha values for the total scale for studies from South America, Asia, and Europe were 0.87, 0.85 and 0.85, respectively (Table 2). Estimates that reported alpha for CIS were mainly from Europe (0.82), followed by Asia (0.86), and South America (0.84). Similarly, estimates that reported alpha for FIS were mainly from Europe (0.72), followed by Asia (0.83), and South America (0.8).

### Language

Two studies have not reported the language of administration explicitly [27, 76]. One study used “Creole” (combination of English and indigenous words) [77]. Pooled estimates for the total scale between publications that used English and other languages questionnaire were similar. However, slightly higher pooled estimates were seen for studies that used English compared to other languages for CIS and FIS subscales respectively (Table 2).

### Quality assessment

The risk of bias was low across all the included publications.

### Sampling

Majority of the estimates have used non-random sampling method [3, 4, 6, 7, 9, 11–16, 18–22, 24–28, 30, 31, 33, 34, 37, 39, 40, 42, 43, 45–49, 51, 52, 54, 56–60, 62–64, 66, 76, 77]. There were no variations in the pooled estimates with different sampling methods for CIS, FIS and total scales.

### Meta-analysis, meta-regression and sub-group analysis

**Fig. 2:**
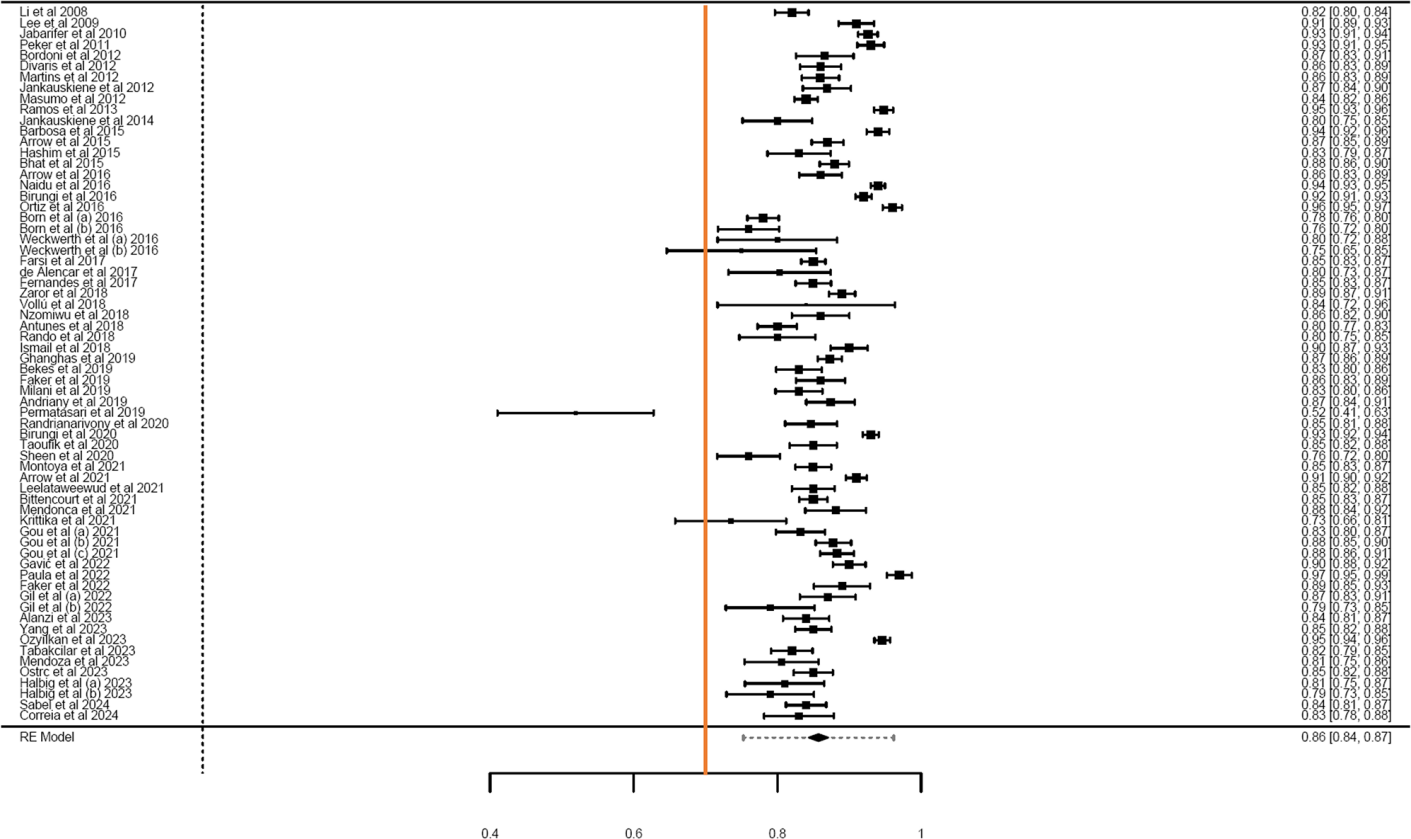
Forest plot showing the cumulative Cronbach’s alpha for the total ECOHIS questionnaire

Meta-analysis included a total sample size of 20,754 (range: 16–1643) from 74 estimates obtained from 66 studies. Cronbach’s α for the total ECOHIS questionnaire was provided by 66 of these estimates which yielded a pooled alpha of 0.86 (95% CI = 0.84–0.87), with high heterogeneity (I^2^ = 95.43%; Q = 1257.07) (Fig. 2). Meta-regression for moderators like publication year, sex, and age (Table 3) showed no significant impact on the Cronbach’s α (*P* > 0.05). Subgroup analysis showed little variation in the pooled estimates for the total scale for continent, language, study setting, study design, and type of sampling (Table 2).

**Fig. 3:**
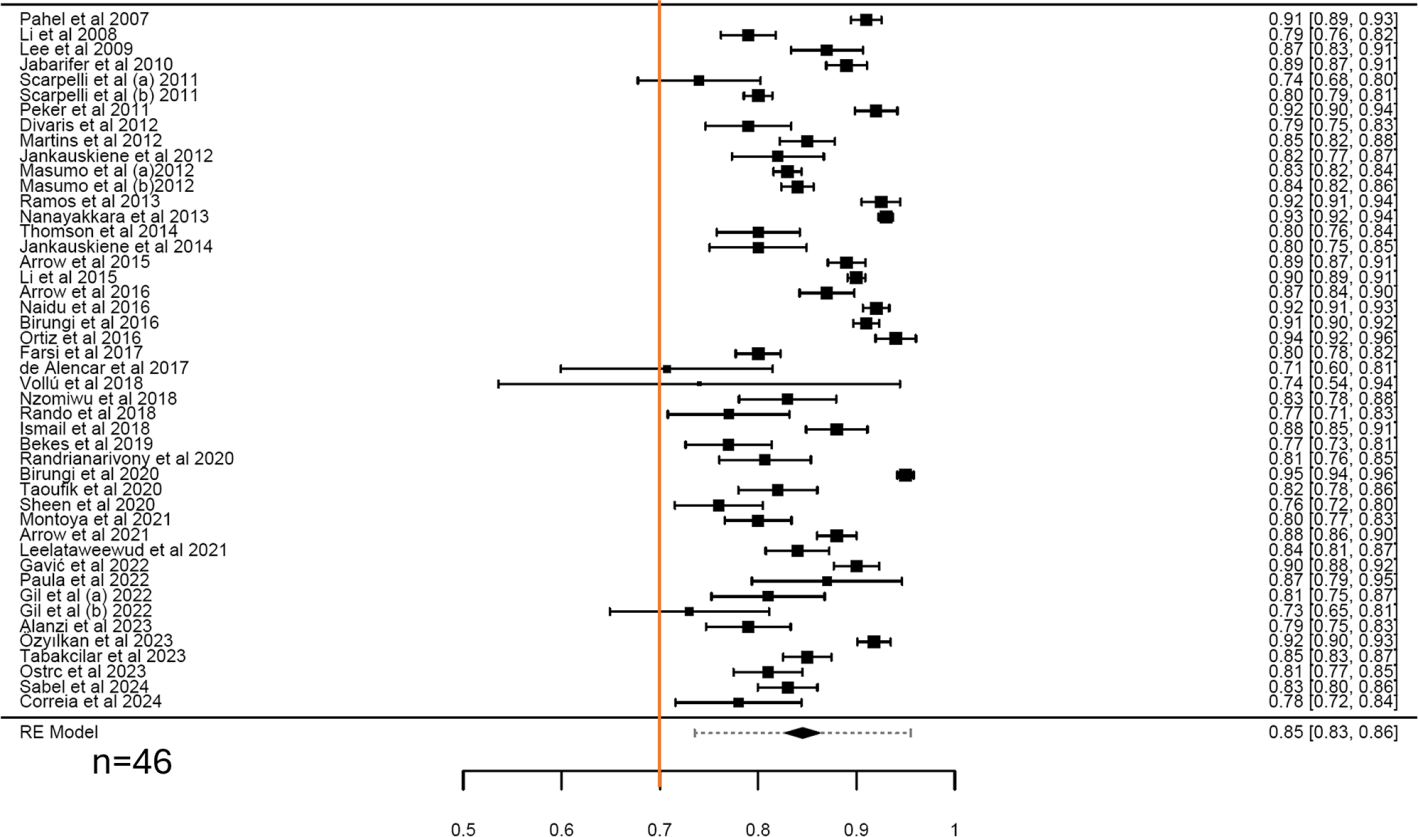
Forest plot showing the cumulative Cronbach’s alpha for CIS

**Fig. 4:**
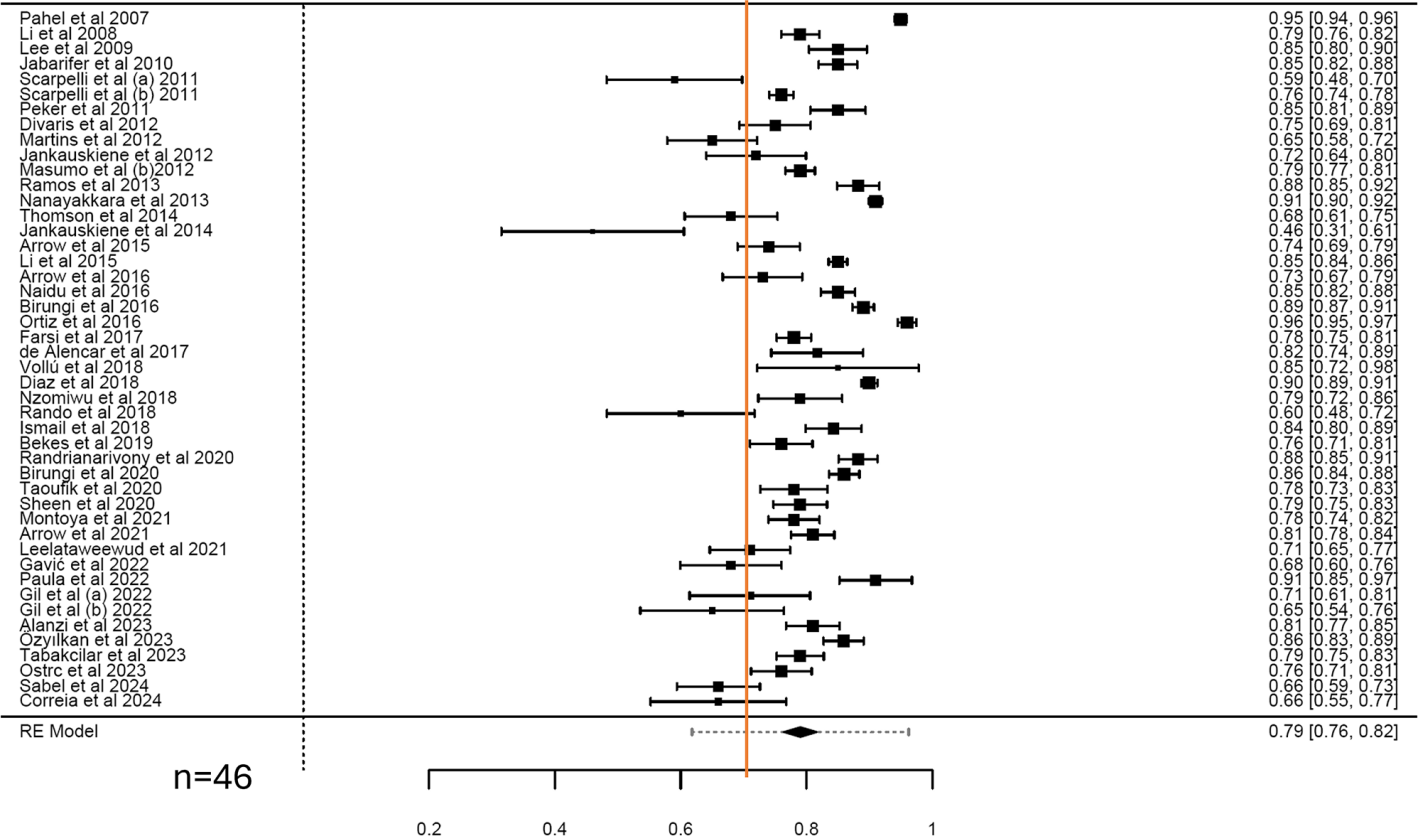
Forest plot showing the cumulative Cronbach’s alpha for FIS

Only 46 estimates were obtained out of the 66 included studies for CIS and FIS scales. The pooled alpha of CIS and FIS were 0.85 (95% CI = 0.83–0.86; I^2^ = 96.52%; Q = 1118.38) (Fig. 3) and 0.79 (95% CI = 0.76–0.82; I^2^ = 97.38%; Q = 1227.64) (Fig. 4) with high heterogeneity. Moderators such as publication year, sex, or age (Table 3) had no significant effect on the pooled Cronbach’s α for both CIS and FIS subscales (*P* > 0.05). Subgroup analysis showed little variation in the pooled estimates for the CIS and FIS for continent, language, study setting, study design, and type of sampling with high heterogeneity across all the categories (Table 2).

### Publication Bias

**Fig. 5:**
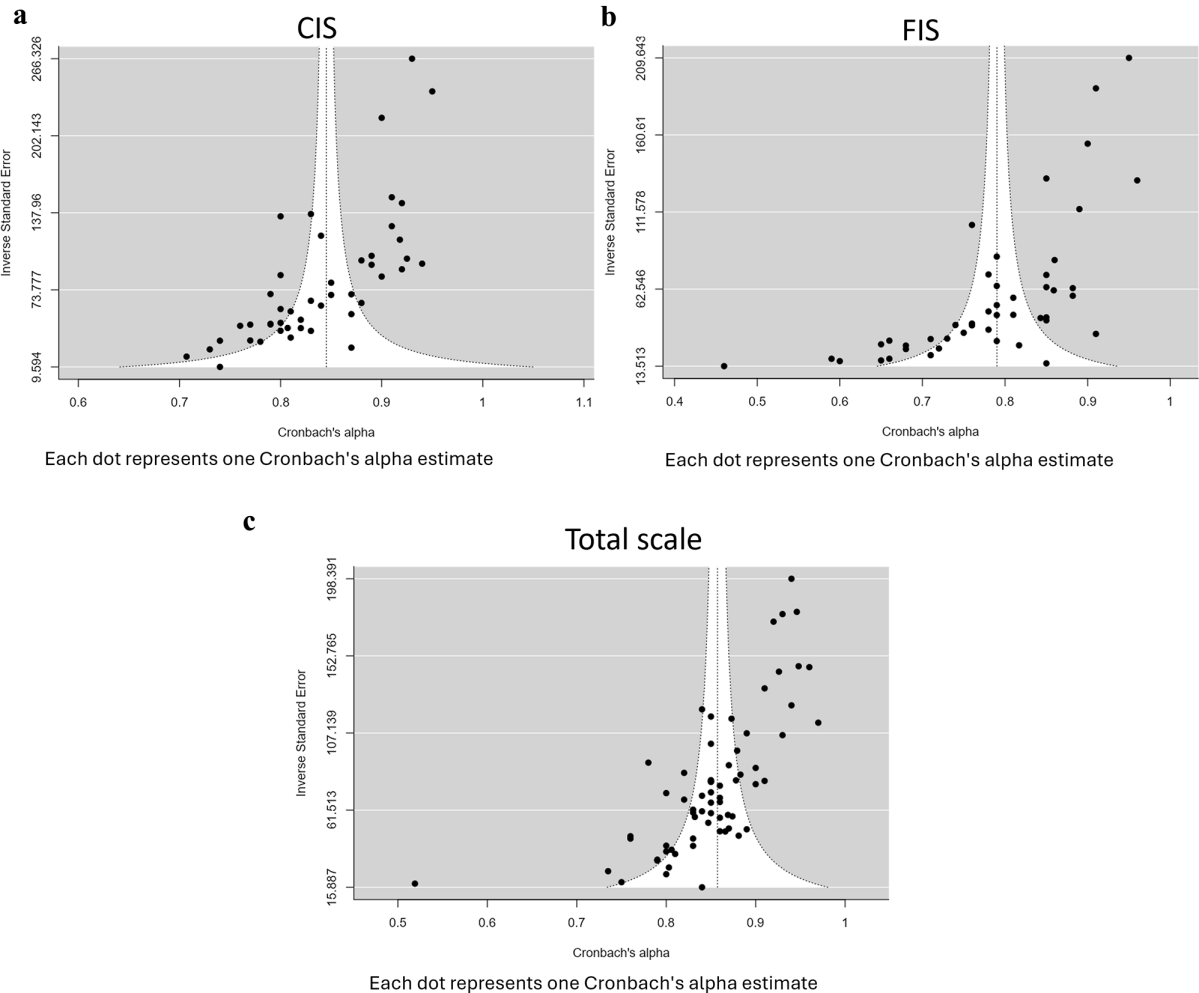
**a** Funnel plot for the assessment of Publication bias for CIS. **b** Funnel plot for the assessment of Publication bias for FIS. **c **Funnel plot for the assessment of Publication bias for total ECOHIS scale

“Egger’s regression” and the “funnel plot” showed publication bias for CIS (coefficient=-6.4; *P* < 0.001) (Fig. 5a), FIS (coefficient=-8.39; *P* < 0.001) (Fig. 5b), and total scale (coefficient=-8.42; *P* < 0.001) (Fig. 5c). This indicated a tendency towards overreporting higher reliability coefficients in the literature.

## Discussion

RGMA summarizes study-level internal consistency scores and estimates variation attributed to potential predictors [79]. ECOHIS questionnaire has been extensively used in various oral conditions, and diverse populations and has been translated into multiple languages for measuring OHRQoL in young children. Most of these studies have reported Cronbach’s α (a widely used metric for internal consistency reliability) above the widely accepted standard which suggested its reliability in multiple cultural and clinical contexts. Therefore, we aimed to the summarize the estimates from various publications that used the ECOHIS questionnaire.

RGMA was performed on various questionnaires that measure OHRQoL [69–72]. However, the pooled reliability estimates of the ECOHIS questionnaire were not reported in the literature. This meta-analysis included 74 estimates from 66 publications, with a total sample size of 20,754. The overall sample size was much lower than the previous RGMA on OIDP questionnaire [71] but higher than SOHO [72] and COHIP [70] questionnaires. It included independent RGMA, meta-regression, and sub-group analysis for the ECOHIS and its subscales. Few publications reported Cronbach’s α for sub-groups due to which higher number of estimates were obtained than the included publications.

The pooled alpha for the total scale was 0.86, which was above the widely accepted benchmark [75]. Similarly, the pooled alpha of CIS (0.85) and FIS (0.79) subscales were also above the benchmark. Few researchers suggested that an alpha of 0.8 and 0.9 is required for research and clinical use respectively [67, 80]. The ECOHIS questionnaire and its subscales have fulfilled these recommendations. The pooled estimates of the total ECOHIS were similar to the COHIP questionnaire (0.87) [70], SOHO child (0.83), and parent versions (0.85) [72] and OIDP questionnaire (0.82) [71]. However, it was higher than the C-OIDP questionnaire (0.73) [69]. High heterogeneity was seen with total and ECOHIS subscales, a similar trend reported in previous RGMA of the OHRQoL questionnaires [69–72]. Meta-regression and sub-group analysis showed that none of the factors evaluated contributed to the high heterogeneity, and a large sample size could be a potential source of heterogeneity along with other factors like cultural variability, different language versions. Meta-analysis of observational studies includes many studies that may have participants with diverse characteristics leading to high heterogeneity. The high heterogeneity in this review might have over or under-estimated the pooled estimates. Future studies should explore the potential sources of heterogeneity using Sensitivity analysis or the use of multivariate meta-analysis models.

Deficiencies in the reporting of Cronbach’s α has led to exclusion of many studies. Among the included publications for full-text screening, a total of 77 studies induced reliability (25.33%). Nevertheless, the results of this RGMA reinforce the internal consistency reliability of the ECOHIS questionnaire and its sub-scales.

There were a few limitations in this RGMA. Only published studies and studies in English were included due to limited resources. Exclusion of non-English studies could have contributed to linguistic publication bias. The inclusion of moderators was limited to those available and accessible from the publications. There was a general lack of consensus in reporting Cronbach’s α because few studies failed to report alpha for total scale or subscales. Few studies didn’t report the mean age, sex, and language of administration due to which the role of these moderators could not be completely explored. This RGMA showed high publication bias likely due to the underrepresentation of lower Cronbach’s α estimates in the literature. Owing to the above limitations, there could be an over or underestimation of the pooled estimates.

Future studies on psychometric research using the ECOHIS questionnaire should report Cronbach’s α for subscales and total questionnaire along with study level characteristics as per the recommended STROBE guidelines.

## Conclusion

The pooled Cronbach’s α for the ECOHIS questionnaire and its subscales were above the acceptable limits and can be used in research and practice. ECOHIS can be used to compare oral health outcomes and oral health impacts among diverse populations.

**Table 1:** Characteristics of the included studies

Author, Year	Continent	*N*	Age(SD)/ Range	Male	Study setting	Language	Study design	Sampling	Number of items		Cronbach’s alpha		
									CIS	FIS	CIS	FIS	Total
Pahel et al. 2007	NA	295	5		S	English	CS	NR	9	4	0.91	0.95	
Li et al. 2008	EU	492	0–5	245	Both	French	CS	NR	9	4	0.79	0.79	0.82
Lee et al. 2009	As	111	49(12)†	60	P	Chinese	CS	NR	9	4	0.87	0.85	0.91
Jabarifer et al. 2010	As	246	4	106	P	Farsi	CS	NR	9	4	0.89	0.85	0.926
Scarpelli et al. (a) 2011	SA	150	4.1(1.2)	77	S	Brazilian	CS	R	10	4	0.74	0.59	
Scarpelli et al. (b) 2011	SA	1643	65.4(3.7)†	843	S	Brazilian	CS	R	9	4	0.8	0.76	
Peker et al. 2011	As	121	5.25(0.43)	56	S	Turkish	CS	NR	9	4	0.92	0.85	0.93
Bordoni et al. 2012	SA	95			S	Spanish	CS	NR	9	4			0.866
Divaris et al. 2012	NA	203	43		S	English	CS	NR	9	4	0.79	0.75	0.86
Martins et al. 2012	SA	247	2–6	123	P	Brazilian Portuguese	CS	R	9	4	0.85	0.65	0.86
Jankauskiene et al. 2012	EU	130	50(13.5)†	57	P	Lithuanian	CS	NR	9	4	0.82	0.72	0.869
Masumo et al. (a)2012	Af	1221	6–36†	616	S	Kiswahili, Luganda	CS	NR	9	4	0.83		
Masumo et al. (b)2012	Af	816	6–36†	414	P	Kiswahili, Luganda	CS	NR	9	4	0.84	0.79	0.84
Ramos et al. 2013	SA	128	3–5	61	S	Spanish	CS	NR	9	4	0.925	0.882	0.948
Nanayakkara et al. 2013	As	784	57.3(6.24) †	383	S	Sinhalese, Tamil	CS	R	9	4	0.93	0.91	
Thomson et al. 2014	Au	195	5.5(1.5)	100	P		CS	NR	9	4	0.8	0.68	
Jankauskiene et al. 2014	EU	144	3.9(0.8)	79	P	Lithuanian	O	NR	9	4	0.8	0.46	0.8
Barbosa et al. 2015	SA	121	5	55	S	Brazilian Portuguese	CS	R	9	4			0.94
Arrow et al. 2015	Au	286	4	230	P	English	O	NR	9	4	0.89	0.74	0.87
Hashim et al. 2015	As	127	4–6.	64	S	Malay	CS	NR	9	4			0.83
Bhat et al. 2015	As	297	4.11	147	S	Malayalam	CS	R	9	4			0.8793
Li et al. 2015	As	1062	3–4.	604	S	Chinese	CS	R	9	4	0.9	0.85	
Arrow et al. 2016	Au	189	3.96, 3.89	111	S		O	NR	9	4	0.87	0.73	0.86
Naidu et al. 2016	SA	309	3.7(0.67)	126	S	English	CS	R	9	4	0.92	0.85	0.94
Birungi et al. 2016	Af	417	4.5(0.6)	208	S	Lumasaba	O	NR	9	4	0.91	0.89	0.92
Ortiz et al. 2016	SA	76	3.96(1.49)	39	P	Brazilian	CS	NR	9	4	0.94	0.96	0.96
Born et al. (a) 2016	NA	844	12.1(3.6) †	428	P	English	O	NR	9	4			0.78
Born et al. (b) 2016	NA	267	11.7(3.6) †	143	P	Spanish	O	NR	9	4			0.76
Weckwerth et al. (a) 2016	SA	50	9.92, 9.66		S	Brazilian Portuguese	CS	NR	10	4			0.8
Weckwerth et al. (b) 2016	SA	50	10.23, 9.87		S	Brazilian Portuguese	CS	NR	10	4			0.75
Farsi et al. 2017	As	668	4.5, 4.6	298	P	Arabic	CS	NR	9	4	0.8	0.78	0.85
de Alencar et al. 2017	SA	66	5.38, 5.29	27	P	Brazilian	CS	NR	9	4	0.707	0.817	0.803
Fernandes et al. 2017	SA	308	29(9.9) †	139	S	Brazilian	CS	R	9	4			0.85
Zaror et al. 2018	SA	302	4(1.1)	163	S	Spanish	CS	NR	9	4			0.89
Vollú et al. 2018	SA	16	3.56(1.31)	10	P	Brazilian	O	NR	9	4	0.74	0.85	0.84
Diaz et al. 2018	SA	643	1–5.	333	S	Spanish	CS	R	9	4		0.9	
Nzomiwu et al. 2018	Af	104	4.3(0.9)	53	P	Nigerian Pidgin English	CS	NR	9	4	0.83	0.79	0.86
Antunes et al. 2018	SA	446	3.63(1.27)	224	S	Brazilian	CS	R	9	4			0.8
Rando et al. 2018	SA	121	46.45†		P	Portuguese	CS	NR	9	4	0.77	0.6	0.8
Ismail et al. 2018	As	130	4.22	65	S	Malay	CS	R	9	4	0.88	0.843	0.9
Ghanghas et al. 2019	As	469	4.05(0.749)	271	S	Hindi	CS	R	9	4			0.873
Bekes et al. 2019	EU	241	3.7(1.2)	113	P	German	CS	NR	9	4	0.77	0.76	0.83
Faker et al. 2019	SA	140	5.5(2.22)	89	P	Brazilian	CS	NR	9	4			0.86
Milani et al. 2019	SA	228	4.31(1.68)	123	P	Brazilian	CS	NR	9	4			0.83
Andriany et al. 2019	As	117	3–5.	62	S	Indonesian	CS	NR	9	4			0.874
Permatasari et al. 2019	As	165	59†		S	Indonesian	CS	NR	9	4			0.519
Randrianarivony et al. 2020	Af	150	3–5.	65	S	Malagasy	CS	R	9	4	0.807	0.882	0.847
Birungi et al. 2020	Af	345	5–7.	175	P	Kiswahili, Luganda	O	NR	6	4	0.95	0.86	0.93
Taoufik et al. 2020	EU	176	4.3		P	Greek	CS	NR	9	4	0.82	0.78	0.85
Sheen et al. 2020	As	251	3–6.	122	S	Taiwanese Mandarin	CS	NR	9	4	0.76	0.79	0.76
Montoya et al. 2021	NA	303	3–5.	155	S	Spanish	CS	R	9	4	0.8	0.78	0.85
Arrow et al. 2021	Au	315	3.6(1.7)	162	S	MIX	O	NR	9	4	0.88	0.81	0.91
Leelataweewud et al. 2021	As	214	42.93(2.86) †	107	S	Thai	CS	R	9	4	0.84	0.71	0.85
Bittencourt et al. 2021	SA	497	4.74(0.42)	247	S	Brazilian	CS	R	9	4			0.85
Mendonca et al. 2021	SA	68	6–8.	33	P	Brazilian	O	NR	9	4			0.881
Krittika et al. 2021	As	100	3–12.	62	P	Tamil	CS	NR	9	4			0.735
Gou et al. (a) 2021	As	204	52.78(8.94) †	113	P	Chinese	O	NR	9	4			0.832
Gou et al. (b) 2021	As	204	52.78(8.94) †	113	P	Chinese	O	NR	9	4			0.878
Gou et al. (c) 2021	As	204	52.78(8.94) †	113	P	Chinese	O	NR	9	4			0.883
Gavić et al. 2022	EU	164	3.89(1.18)	89	P	Croatian	CS	NR	9	4	0.9	0.68	0.9
Paula et al. 2022	SA	27	8.44(3.17)	15	P	Brazilian	O	NR	9	4	0.87	0.91	0.97
Faker et al. 2022	SA	68	4.47, 4.21	36	P	Brazilian	CS	NR	9	4			0.89
Gil et al. (a) 2022	EU	96	4–11.		P	Norwegian	CS	NR	9	4	0.81	0.71	0.87
Gil et al. (b) 2022	EU	98	4–11.		P	Norwegian	CS	NR	9	4	0.73	0.65	0.79
Alanzi et al. 2023	As	207	48–71	106	S	Arabic	CS	R	9	4	0.79	0.81	0.84
Yang et al. 2023	As	300	4.1(0.7)	163	P	Chinese	CS	NR	9	4			0.85
Özyılkan et al. 2023	EU	204	2–18.	94	P	Turkish	CS	NR	9	4	0.918	0.859	0.946
Tabakcilar et al. 2023	As	324	3.55(0.75)	205	P	Turkish	CS	NR	9	4	0.85	0.79	0.82
Mendoza et al. 2023	SA	120	3–5.	58	P	Spanish	CS	NR	9	4			0.806
Ostrc et al. 2023	EU	255	4.8(0.8)	130	S	Slovenian	CS	NR	9	4	0.81	0.76	0.85
Halbig et al. (a) 2023	EU	101	7.6, 7.8	27	P	Norwegian	O	NR	9	4			0.81
Halbig et al. (b) 2023	EU	101	9.7, 9.8	27	P	Norwegian	O	NR	9	4			0.79
Sabel et al. 2024	EU	274	4	145	P	Swedish	CS	NR	9	4	0.83	0.66	0.84
Correia et al. 2024	EU	104	4.1(0.8)	47	S	Portuguese	CS	NR	9	4	0.78	0.66	0.83

N: sample size; NR: Non-random; R: random; Eu: Europe; SA: South America; NA: North America; Af: Africa; As: Asia; Au: Australia; CS: Cross-sectional; O: Others (Trial or follow-up study); P: Patients; S: School or Community

**Table 2:** Subgroup analysis of the cumulative cronbach’s alpha of CIS, FIS and total ECOHIS questionnaire for various categorical predictors

	CIS					FIS					Total Scale				
	*N*	Estimate	SE	95% CI	I^2^	*N*	Estimate	SE	95% CI	I^2^	*N*	Estimate	SE	95% CI	I^2^
Continent															
Africa	6(13.04)	0.86	0.02	0.82–0.91	97.95	5(10.87)	0.85	0.02	0.81–0.89	91.89	5(7.58)	0.88	0.02	0.84–0.92	96.06
Asia	11(23.91)	0.86	0.02	0.83–0.89	96.97	11(23.91)	0.83	0.02	0.79–0.86	92.36	19(28.79)	0.85	0.16	0.82–0.88	96.63
Australia	4(8.7)	0.86	0.02	0.83–0.9	87.62	4(8.7)	0.75	0.03	0.69–0.8	76.15	3(4.55)	0.88	0.02	0.85–0.91	83.82
Europe	12(26.09)	0.82	0.02	0.79–0.85	88.5	12(26.09)	0.72	0.03	0.67–0.77	88.02	14(21.21)	0.85	0.01	0.82–0.87	88.79
North America	3(6.52)	0.84	0.04	0.76–0.91	94.97	3(6.52)	0.83	0.06	0.71–0.95	97.46	4(6.06)	0.81	0.02	0.77–0.86	91.54
South America	10(21.74)	0.84	0.03	0.77–0.89	97.18	11(23.91)	0.8	0.04	0.73–0.88	98.57	21(31.82)	0.87	0.01	0.84–0.9	95.92
Language															
English	4(9.09)	0.88	0.03	0.83–0.94	97.06	4(9.09)	0.83	0.05	0.73–0.92	97.79	4(6.15)	0.86	0.03	0.8–0.93	97.82
Others	40(90.91)	0.84	0.01	0.82–0.86	96.55	40(90.91)	0.79	0.02	0.76–0.82	97.06	61(93.85)	0.86	0.01	0.84–0.87	95.22
Study setting															
School or community	21(45.65)	0.85	0.01	0.83–0.88	97.52	21(45.65)	0.82	0.02	0.78–0.85	97.81	28(42.42)	0.9	0.01	0.84–0.88	96.63
Others	25(54.35)	0.84	0.01	0.82–0.86	94.56	25(54.35)	0.77	0.02	0.73–0.81	95.3	38(57.58)	0.86	0.01	0.84–0.87	94.4%
Study Design															
Cross-sectional	38(82.61)	0.84	0.01	0.82–0.86	96.16	38(82.61)	0.79	0.01	0.76–0.82	97.31	50(75.76)	0.86	0.01	0.84–0.87	94.97
Others	8(17.39)	0.88	0.02	0.85–0.92	94.73	8(17.39)	0.79	0.05	0.7–0.88	97.46	16(24.24)	0.86	0.02	0.83–0.89	96.36
Sampling															
Random	11(23.91)	0.85	0.02	0.81–0.88	97.75	12(26.09)	0.8	0.03	0.75–0.85	98.39	13(19.7)	0.87	0.01	0.85–0.89	93.16
Non-random	35(76.09)	0.85	0.01	0.83–0.87	95.62	34(73.91)	0.79	0.02	0.76–0.82	96.41	53(80.3)	0.85	0.01	0.84–0.87	95.77

N: number of estimates; SE: standard error; CI: Confidence Interval

**Table 3:** Moderator analysis using Meta-regression for CIS, FIS and total ECOHIS questionnaire

	CIS					FIS					Total scale				
	*N*	Coefficient	*p*-value	95%CI	*R* ^2^	*N*	Coefficient	*p*-value	95%CI	*R* ^2^	*N*	Coefficient	*p*-value	95%CI	*R* ^2^
Publication year	46	-0.002	0.31	-0.005-0.002	0	46	-0.002	0.329	-0.008-0.003	0.32	66	-0.002	0.2	-0.006-0.001	0.93
Sex	40	0.01	0.4	-0.01-0.04	0	40	0.019	0.436	-0.053-0.023	0	66	-0.001	0.959	-0.022-0.021	0
Mean age	32	0.002	0.89	-0.02-0.03	0	32	0.03	0.081	-0.004-0.069	6.47	48	0	0.999	-0.01-0.01	0

## Electronic supplementary material

Below is the link to the electronic supplementary material.

Supplementary Material 1

Supplementary Material 2

## Data Availability

All supporting data for this review are included within the manuscript.

## Abbreviations

CI: Confidence Interval
COSMIN: COnsensus-based Standards for the selection of health Measurement Instruments
CIS: Child Impact Section
C-OIDP: Child– Oral Impacts on Daily Performances
COHIP: Child Oral Health Impact Profile
ECOHIS: Early Childhood Oral Health Impact scale
FIS: Family Impact Section
OHRQoL: Oral Health Related Quality of Life
OIDP: Oral Impacts on Daily Performances
RGMA: Reliability Generalization Meta-Analysis
REGEMA: Reporting Quality of Reliability Generalization Meta-Analyses
SOHO: Scale of Oral Health Outcomes

## References

[1] Min SN, Duangthip D, Gao SS, Detsomboonrat P. Quality of the adaptation procedures and psychometric properties of the scale of oral health outcomes for 5-year-old children (SOHO-5): a systematic review. Qual Life Res. 2023;32:1537–47. 10.1007/S11136-022-03280-2. 36273047 10.1007/s11136-022-03280-2

[2] Barbosa TS, Gavião MB. Oral health-related quality of life in children: part I. How well do children know themselves? A systematic review. Int J Dent Hyg. 2008;6:93–9. 10.1111/J.1601-5037.2007.00276.X. 18412720 10.1111/j.1601-5037.2007.00276.x

[3] Pahel BT, Rozier RG, Slade GD. Parental perceptions of children’s oral health: the early childhood oral health impact scale (ECOHIS). Health Qual Life Outcomes. 2007;5:6. 10.1186/1477-7525-5-6. 17263880 10.1186/1477-7525-5-6 PMC1802739

[4] Lee GHM, McGrath C, Yiu CKY, King NM. Translation and validation of a Chinese Language version of the early childhood oral health impact scale (ECOHIS). Int J Paediatr Dent. 2009;19:399–405. 10.1111/j.1365-263X.2009.01000.x. 19811551 10.1111/j.1365-263X.2009.01000.x

[5] Martins-Júnior PA, Ramos-Jorge J, Paiva SM, Marques LS, Ramos-Jorge ML. Validations of the Brazilian version of the early childhood oral health impact scale (ECOHIS). Cad Saude Publica. 2012;28:367–74. 10.1590/S0102-311X2012000200015. 22331162 10.1590/s0102-311x2012000200015

[6] López Ramos RP, García Rupaya CR, Villena-Sarmiento R, Bordoni NE. Cross cultural adaptation and validation of the early childhood health impact scale (ECOHIS) in Peruvian preschoolers. Acta Odontol Latinoam. 2013;26:60–7. 24303728

[7] Arrow P, Klobas E. Evaluation of the early childhood oral health impact scale in an Australian preschool child population. Aust Dent J. 2015;60:375–81. 10.1111/ADJ.12236. 25324159 10.1111/adj.12236

[8] Fernandes IB, Ramos-Jorge J, Ramos-Jorge ML, Bönecker M, Abanto J, Marques LS, Paiva SM. Correlation and comparative analysis of discriminative validity of the scale of oral health outcomes for Five-Year-Old children (SOHO-5) and the early childhood oral health impact scale (ECOHIS) for dental caries. BMC Oral Health. 2015;15. 10.1186/S12903-015-0021-Y. 10.1186/s12903-015-0021-y PMC4359477 25881305

[9] Hashim AN, Yusof ZYM, Esa R. The Malay version of the Early Childhood Oral Health Impact Scale (Malay-ECOHIS)--assessing validity and reliability. Health Qual Life Outcomes 2015;13. 10.1186/S12955-015-0386-2 10.1186/s12955-015-0386-2 PMC4660630 26607665

[10] Nanayakkara V, Renzaho A, Oldenburg B, Ekanayake L. Ethnic and socio-economic disparities in oral health outcomes and quality of life among Sri Lankan preschoolers: A cross-sectional study. Int J Equity Health. 2013;12:1–9. 10.1186/1475-9276-12-89/TABLES/4. 24228941 10.1186/1475-9276-12-89 PMC3830507

[11] Peker K, Uysal Ö, Bermek G. Cross - cultural adaptation and preliminary validation of the Turkish version of the early childhood oral health impact scale among 5-6-year-old children. Health Qual Life Outcomes. 2011;9. 10.1186/1477-7525-9-118. 10.1186/1477-7525-9-118 PMC3310831 22192577

[12] De Alencar NA, Leão CS, Leão ATT, Luiz RR, Fonseca-Gonçalves A, Maia LC. Sleep Bruxism and anxiety impacts in quality of life related to oral health of Brazilian children and their families. J Clin Pediatr Dent. 2017;41:179–85. 10.17796/1053-4628-41.3.179. 28422599 10.17796/1053-4628-41.3.179

[13] Rando GM, Jorge PK, Vitor LLR, Carrara CFC, Soares S, Silva TC, Rios D, Machado MAAM, Gavião MB, Oliveira TM. Oral health-related quality of life of children with oral clefts and their families. J Appl Oral Sci. 2018;26. 10.1590/1678-7757-2017-0106. 10.1590/1678-7757-2017-0106 PMC5777410 29412367

[14] Huamán Mendoza AA, Pinedo Tellez KS, Rodrigues de Almeida Silva C, Tello Guerrero YG, Calle Lopez P, García Rupaya CR, Valdez Jurado FR. Factors associated with oral health relatedquality of life in preschoolers from an Andean community. Rev Estomatológica Hered. 2023;33:26–33. 10.20453/reh.v33i1.4431.

[15] Zaror C, Atala-Acevedo C, Espinoza-Espinoza G, Muñoz-Millán P, Muñoz S, Martínez-Zapata MJ, Ferrer M. Cross-cultural adaptation and psychometric evaluation of the early childhood oral health impact scale (ECOHIS) in Chilean population. Health Qual Life Outcomes. 2018;16. 10.1186/S12955-018-1057-X. 10.1186/s12955-018-1057-x PMC6296046 30554568

[16] Milani AJ, Fonseca Alves N, Martins Do Espiroto-Santo T, Gonçalves Ribeiro L, Ammari MM, Antunes LS, Alves Antunes LA. Impact of traumatic dental injuries on oral Health-Related quality of life of preschool children and their families attending a dental trauma care program. Port J Public Heal. 2019;37:19–25. 10.1159/000501525.

[17] Díaz S, Vélez MP, Martínez LM, Ramos K, Boneckër M, Martins Paiva S, Abanto J. Parental perceptions of impact of oral disorders on Colombian schoolchildren’s oral healthrelated quality of life. Acta Odontol Latinoam. 2018;31:82–90. 30383071

[18] Tabakcilar D, Peker K, Yilmaz DO, Kasimoglu Y, Tuna-Ince EB, Gencay K, Seymen F. Evaluation of the predictors of oral health-related quality of life among 3-5-year-old children with dental trauma. Braz Oral Res. 2023;36. 10.1590/1807-3107BOR-2022.VOL36.0140. 10.1590/1807-3107bor-2022.vol36.0140 36651387

[19] Masumo R, Bardsen A, Mashoto K, Åstrøm AN. Child- and family impacts of infants’ oral conditions in Tanzania and Uganda- a cross sectional study. BMC Res Notes. 2012;5. 10.1186/1756-0500-5-538. 10.1186/1756-0500-5-538 PMC3532836 23016603

[20] Jabarifar SE, Golkari A, IJadi MH, Jafarzadeh M, Khadem P. Validation of a Farsi version of the early childhood oral health impact scale (F-ECOHIS). BMC Oral Health. 2010;10:4. 10.1186/1472-6831-10-4. 20367888 10.1186/1472-6831-10-4 PMC2858088

[21] Ortiz FR, dos Santos MD, Landenberger T, Emmanuelli B, Agostini BA, Ardenghi TM. Comparison of Face-To-Face interview and telephone methods of administration on the ecohis scores. Braz Dent J. 2016;27:613–8. 10.1590/0103-6440201601134. 27982244 10.1590/0103-6440201601134

[22] Halbig JM, Jönsson B, Gil EG, Åstrøm AN, Rypdal V, Frid P, Augdal TA, Fischer J, Cetrelli L, Rygg M, Lundestad A, Tylleskär K, Nordal E. Oral health-related quality of life, impaired physical health and orofacial pain in children and adolescents with juvenile idiopathic arthritis - a prospective multicenter cohort study. BMC Oral Health. 2023;23. 10.1186/S12903-023-03510-0. 10.1186/s12903-023-03510-0 PMC10662257 37986155

[23] Bhat SG, Sivaram R. Psychometric properties of the Malayalam version of ECOHIS. J Indian Soc Pedod Prev Dent. 2015;33:234–8. 10.4103/0970-4388.160398. 26156279 10.4103/0970-4388.160398

[24] Farsi NJ, El-Housseiny AA, Farsi DJ, Farsi NM. Validation of the Arabic version of the early childhood oral health impact scale (ECOHIS). BMC Oral Health. 2017;17. 10.1186/S12903-017-0353-X. 10.1186/s12903-017-0353-x PMC5331632 28245876

[25] Andriany P, Pintauli S, Lubis R, Rahardjo A. Trans cultural adaptation and validation Indonesia version of early childhood oral health impact scale (I-ECOHIS). J Int Dent Med Res. 2019;12:1591–6.

[26] Jankauskiene B, Narbutaite J, Kubilius R, Gleiznys A. Adaptation and validation of the early childhood oral health impact scale in Lithuania. Stomatologija. 2012;14:108–13. 23455979

[27] Thomson WM, Foster Page LA, Malden PE, Gaynor WN, Nordin N. Comparison of the ECOHIS and short-form P-CPQ and FIS scales. Health Qual Life Outcomes. 2014;12. 10.1186/1477-7525-12-36. 10.1186/1477-7525-12-36 PMC3984713 24618408

[28] Jankauskiene B, Virtanen JI, Kubilius R, Narbutaite J. Oral health-related quality of life after dental general anaesthesia treatment among children: a follow-up study. BMC Oral Health 2014;14. 10.1186/1472-6831-14-81 10.1186/1472-6831-14-81 PMC4090347 24984901

[29] Randrianarivony J, Ravelomanantsoa JJ, Razanamihaja N. Evaluation of the reliability and validity of the early childhood oral health impact scale (ECOHIS) questionnaire translated into Malagasy. Health Qual Life Outcomes. 2020;18:1–11. 10.1186/S12955-020-01296-1/TABLES/7. 10.1186/s12955-020-01296-1 PMC7038613 32093708

[30] Divaris K, Lee JY, Baker AD, Vann WF. Caregivers’ oral health literacy and their young children’s oral health-related quality-of-life. Acta Odontol Scand. 2012;70:390–7. 10.3109/00016357.2011.629627. 22150574 10.3109/00016357.2011.629627 PMC3305855

[31] Gou C, Wang Y, Yang R, Huang R, Zhang Q, Zou J. Oral health-related quality of life and parental anxiety in Chinese children undergoing dental general anesthesia: a prospective study. BMC Oral Health. 2021;21. 10.1186/S12903-021-01994-2. 10.1186/s12903-021-01994-2 PMC8711163 34961505

[32] Alanzi A, Husain F, Husain H, Hanif A, Baskaradoss J. Does the severity of untreated dental caries of preschool children influence the oral health-related quality of life? BMC Oral Health. 2023;23. 10.1186/S12903-023-03274-7. 10.1186/s12903-023-03274-7 PMC10416462 37563589

[33] Faker K, Tostes MA, de Paula VAC. Oral health-related quality of life among autistic children compared to children without autism in Brazil. Int J Clin Dent. 2022;15:27–37.

[34] De Paula VAC, Faker K, Bendo CB, Tostes MA. Responsiveness of the B-ECOHIS to detect changes in OHRQoL following dental treatment of children with autism spectrum disorder.Braz Oral Res 2022;36. 10.1590/1807-3107BOR-2022.VOL36.0079. 10.1590/1807-3107bor-2022.vol36.0079 35703705

[35] Scarpelli AC, Oliveira BH, Tesch FC, Leão AT, Pordeus IA, Paiva SM. Psychometric properties of the Brazilian version of the early childhood oral health impact scale (B-ECOHIS). BMC Oral Health. 2011;11. 10.1186/1472-6831-11-19. 10.1186/1472-6831-11-19 PMC3125235 21668959

[36] Fernandes IB, Pereira TS, Souza DS, Ramos-Jorge J, Marques LS, Ramos-Jorge ML. Severity of dental caries and quality of life for toddlers and their families. Pediatr Dent. 2017;39:118–23. 28390461

[37] Vollú AL, Requejo Da Costa MDEP, Cople Maia L, Fonseca-Gonçalves A. Evaluation of oral Health-Related quality of life to assess dental treatment in preschool children with early childhood caries: A preliminary study. J Clin Pediatr Dent. 2018;42:37–44. 10.17796/1053-4628-42.1.7. 28937896 10.17796/1053-4628-42.1.7

[38] Antunes LAA, Ornellas G, Fraga RS, Antunes LS. Oral health outcomes: the association of clinical and socio-dental indicators to evaluate dental caries in preschool children. Cien Saude Colet. 2018;23:491–500. 10.1590/1413-81232018232.21022015. 29412407 10.1590/1413-81232018232.21022015

[39] Faker K, Tostes MA, de Paula VAC. Impact of untreated dental caries on oral health-related quality of life of children with special health care needs. Braz Oral Res 2019;32.10.1590/1807-3107BOR-2018.VOL32.0117. 10.1590/1807-3107BOR-2018.vol32.0117 30892372

[40] Mendonça JGA, Almeida RF, Leal SC, de Macedo Bernardino Í, Hilgert LA, Ribeiro APD.Impact of dental treatment on the oral health-related quality of life of Brazilian schoolchildren. Braz Oral Res 2021;35. 10.1590/1807-3107BOR-2021.VOL35.0125. 10.1590/1807-3107bor-2021.vol35.0125 34878080

[41] Bittencourt JM, Martins LP, Paiva SM, Pordeus IA, Martins-Júnior PA, Bendo CB. Early childhood caries and oral health-related quality of life of Brazilian children: does parents’ resilience act as moderator? Int J Paediatr Dent. 2021;31:383–93. 10.1111/IPD.12727. 32941667 10.1111/ipd.12727

[42] Weckwerth SAM, Weckwerth GM, Ferrairo BM, Chicrala GM, Ambrosio AMN, Toyoshima GHL, Bastos JRM, Pinto EC, Velasco SRM, Bastos RS. Parents’ perception of dental caries in intellectually disabled children. Spec Care Dentist. 2016;36:300–6. 10.1111/SCD.12191. 27420288 10.1111/scd.12191

[43] Correia C, Graça SR, Mendes S. Early childhood oral health impact scale: psychometric evaluation in Portuguese preschoolers. Acta Stomatol Croat. 2024;58:39–51. 10.15644/ASC58/1/4. 38562224 10.15644/asc58/1/4 PMC10981910

[44] Li MY, Zhi QH, Zhou Y, Qiu RM, Lin HC. Impact of early childhood caries on oral health-related quality of life of preschool children. Eur J Paediatr Dent. 2015;16:65–72. 25793957

[45] Yang L, Zhao S, Zhu Y, Lai G, Wang J. Oral health-related quality of life and associated factors among a sample from East China with severe early childhood caries: a cross-sectional study. BMC Oral Health. 2023;23. 10.1186/S12903-023-03560-4. 10.1186/s12903-023-03560-4 PMC10629075 37936111

[46] Gavic L, Tadin A, Guiin J, Jerkovic D, Hrvatin S, Sidhu SK. Relationship between early childhood oral health impact scale, child’s dental status and parental psychological profiles. Psychiatr Danub. 2022;34:168–72. 36752257

[47] Li S, Veronneau J, Allison PJ. Validation of a French Language version of the early childhood oral health impact scale (ECOHIS). Health Qual Life Outcomes. 2008;6. 10.1186/1477-7525-6-9. 10.1186/1477-7525-6-9 PMC2245912 18211711

[48] Bekes K, Omara M, Safar S, Stamm T. The German version of early childhood oral health impact scale (ECOHIS-G): translation, reliability, and validity. Clin Oral Investig. 2019;23:4449–54. 10.1007/S00784-019-02893-1. 30993536 10.1007/s00784-019-02893-1

[49] Taoufik K, Divaris K, Kavvadia K, Koletsi-Kounari H, Polychronopoulou A. Development and validation of the Greek version of the early childhood oral health impact scale (ECOHIS). Open Dent J. 2020;14:88–96. 10.2174/1874210602014010088.

[50] Ghanghas M, Manjunath B, Kumar A, Shyam R, Phogat R, Panghal V. Validation of the Hindi version of the early childhood oral health impact scale among 3-5-year-old preschool children in Rohtak city, Haryana. J Indian Soc Pedod Prev Dent. 2019;37:333–8. 10.4103/JISPPD.JISPPD_128_18. 31710006 10.4103/JISPPD.JISPPD_128_18

[51] Permatasari RF, Setiawati F, Badruddin IA. Association between early childhood caries and oral health-related quality of life using ecohis instrument. J Int Dent Med Res. 2019;12:1017–21.

[52] Birungi N, Fadnes LT, Engebretsen IMS, Lie SA, Tumwine JK, Åstrøm AN. Caries experience and oral health related quality of life in a cohort of Ugandan HIV-1 exposed uninfected children compared with a matched cohort of HIV unexposed uninfected children. BMC Public Health. 2020;20:1–12. 10.1186/S12889-020-08564-1/TABLES/5. 32228542 10.1186/s12889-020-08564-1 PMC7106612

[53] Basir L, Shayeste M, Imani Z, Haghighizade MH, Kartalai MM, Heidari MA. Effects of oral health in quality of life among children after dental treatment under general anesthesia. Int J Pharm Technol. 2016;8:11643–51.

[54] Birungi N, Fadnes LT, Nankabirwa V, Tumwine JK, Åstrøm AN. Caretaker’s caries experience and its association with early childhood caries and children’s oral health-related quality of life: A prospective two-generation study. Acta Odontol Scand. 2016;74:605–12. 10.1080/00016357.2016.1225981. 27571601 10.1080/00016357.2016.1225981

[55] Syafiqah Ismail N, Murshidah Abdul Ghani N, Supaat S, Fazwan Kharuddin A, Dewi Ardini Y. The early childhood oral health impact scale (ECOHIS): assessment tool in oral health related quality of life. J Int Dent Med Res. 2018;11:162–8.

[56] Nzomiwu CL, Sote EO, Oredugba FA. Translation and validation of the Nigerian pidgin english version of the early childhood oral health impact scale (NAIJA ECOHIS). West Afr J Med. 2018;35:102–8. 30027995

[57] Gil EG, Skeie MS, Halbig J, Jönsson B, Lie SA, Rygg M, Fischer J, Rosén A, Bletsa A, Luukko K, Shi XQ, Frid P, Cetrelli L, Tylleskär K, Rosendahl K, Åstrøm AN. Oral health-related quality of life in 4-16-year-olds with and without juvenile idiopathic arthritis. BMC Oral Health. 2022;22. 10.1186/S12903-022-02400-1. 10.1186/s12903-022-02400-1 PMC9450232 36068497

[58] Likar Ostrc L, Frankovič S, Pavlič A. The development and evaluation of the Slovenian version of the early childhood oral health impact scale (ECOHIS-SVN). Zdr Varst. 2023;62:173–81. 10.2478/SJPH-2023-0025. 37799415 10.2478/sjph-2023-0025 PMC10549250

[59] Bordoni N, Ciaravino O, Zambrano O, Villena R, Beltran-Aguilar E, Squassi A. Early childhood oral health impact scale (ECOHIS). Translation and validation in Spanish Language. Acta Odontol Latinoam. 2012;25:270–8. 23798073

[60] Born CD, Divaris K, Zeldin LP, Rozier RG. Influences on preschool children’s oral health-related quality of life as reported by english and Spanish-speaking parents and caregivers. J Public Health Dent. 2016;76:276–86. 10.1111/JPHD.12152. 26990804 10.1111/jphd.12152

[61] Montoya ALB, Knorst JK, Uribe IMP, González RAB, Ardenghi TM, Sánchez CCA. Cross-cultural adaptation and psychometric properties of the Mexican version of the early childhood oral health impact scale (ECOHIS). Health Qual Life Outcomes. 2021;19. 10.1186/S12955-021-01747-3. 10.1186/s12955-021-01747-3 PMC7981842 33743730

[62] Sabel N, Ylander LO, Ståhlberg SE, Robertson A. Dental caries and oral health-related quality of life in Preschoolers - introducing the Swedish version of the early childhood oral health impact scale (ECOHIS). Acta Odontol Scand. 2024;83:47–53. 10.1080/00016357.2023.2287235. 38032108 10.1080/00016357.2023.2287235 PMC11302645

[63] Sheen MH, Hsiao SY, Huang S, Te. Translation and validation of Taiwanese version of the early childhood oral health impact scale (ECOHIS). J Dent Sci. 2020;15:513–8. 10.1016/J.JDS.2020.05.029. 10.1016/j.jds.2020.05.029 PMC7816025 33505624

[64] Krittika R, Ramakrishnan M. Development and validation of Tamil version of ecohis scale T ECOHIS: A cross sectional study. J Res Med Dent Sci. 2021;9:187–90.

[65] Leelataweewud P, Jirarattanasopha V, Ungchusak C, Vejvithee W. Psychometric evaluation of the Thai version of the early childhood oral health impact scale (Th-ECOHIS): a cross sectional validation study. BMC Oral Health. 2021;21:1–11. 10.1186/S12903-020-01332-Y/TABLES/6. 33573657 10.1186/s12903-020-01332-y PMC7879657

[66] Özyılkan D, Tosun Ö, İslam A. The impact of Anemia-Related early childhood caries on parents’ and children’s quality of life. Med (Kaunas). 2023;59. 10.3390/MEDICINA59030521. 10.3390/medicina59030521 PMC10052039 36984522

[67] Nunnally JC, Bernstein IH. Psychometric theory. McGraw-Hill; 1994.

[68] Buczynski AK, Castro GF, Leão AT, Souza IPR. Impact of oral health on the quality of life of 3-6-years old HIV-infected children. Quality of life in HIV + children. Eur J Paediatr Dent. 2011;12:236–8. 21668276

[69] Pentapati KC, Yeturu SK, Siddiq H. A reliability generalization meta-analysis of child oral impacts on daily performances (C - OIDP) questionnaire. J Oral Biol Craniofac Res. 2020;10:776–81. 10.1016/J.JOBCR.2020.10.017. 33145155 10.1016/j.jobcr.2020.10.017 PMC7596296

[70] Pentapati KC, Chenna D, Kumar VS, Kumar N, Kumar S. Child oral health impact profile questionnaire: A reliability generalization Meta-analysis of cronbach’s alpha. Int J Clin Pediatr Dent. 2024;17:1193–8. 10.5005/jp-journals-10005-2973. 39650288 10.5005/jp-journals-10005-2973 PMC11617429

[71] Pentapati KC, Chenna D, Kumar VS, Kumar N. Reliability generalization meta-analysis of cronbach’s alpha of the oral impacts on daily performance (OIDP) questionnaire. BMC Oral Health. 2025;25:220. 10.1186/s12903-025-05496-3. 39934795 10.1186/s12903-025-05496-3 PMC11817562

[72] Pentapati K, Chenna D, Kumar VS, Kumar N. Scale of oral health outcomes (SOHO) for children: A reliability generalization Meta-analysis of cronbach’s alpha. Int J Clin Pediatr Dent 2025;18:Ahead of Print. 10.5005/jp-journals-10005-3156 PMC12486633 41041010

[73] Mokkink LB, de Vet HCW, Prinsen CAC, Patrick DL, Alonso J, Bouter LM, Terwee CB. COSMIN risk of Bias checklist for systematic reviews of Patient-Reported outcome measures. Qual Life Res. 2018;27:1171–9. 10.1007/s11136-017-1765-4. 29260445 10.1007/s11136-017-1765-4 PMC5891552

[74] JAMOVI. jamovi - open statistical software for the desktop and cloud. Software 2021:1–1. https://www.jamovi.org/ (accessed October 6, 2023).

[75] Jum C. Nunnally. Psychometric theory. 2nd ed. New York: McGraw-Hill; 1978.

[76] Arrow P. Responsiveness and sensitivity of the early childhood oral health impact scale to primary dental care for early childhood caries. Community Dent Oral Epidemiol. 2016;44:1–10. 10.1111/CDOE.12183. 26179655 10.1111/cdoe.12183

[77] Arrow P, Brennan D, Mackean T, McPhee R, Kularatna S, Jamieson L. Evaluation of the ECOHIS and the CARIES-QC among an Australian aboriginal population. Qual Life Res. 2021;30:531–42. 10.1007/S11136-020-02646-8. 32974881 10.1007/s11136-020-02646-8

[78] Naidu R, Nunn J, Donnelly-Swift E. Oral health-related quality of life and early childhood caries among preschool children in Trinidad. BMC Oral Health. 2016;16:128. 10.1186/S12903-016-0324-7. 27923355 10.1186/s12903-016-0324-7 PMC5142136

[79] Greco LM, O’Boyle EH, Cockburn BS, Yuan Z. Meta-Analysis of coefficient alpha: A reliability generalization study. J Manag Stud. 2018;55:583–618. 10.1111/JOMS.12328.

[80] Charter RA. A breakdown of reliability coefficients by test type and reliability method, and the clinical implications of low reliability. J Gen Psychol. 2003;130:290–304. 10.1080/00221300309601160. 12926514 10.1080/00221300309601160

