# Supplementary material for: Early Childhood Oral Health Impact Scale (ECOHIS) questionnaire: reliability generalization meta-analysis of Cronbach’s alpha
Source: BMC Oral Health. 2025 Jun 9;25:947. doi: 10.1186/s12903-025-06342-2 (PMC12150495; doi:10.1186/s12903-025-06342-2)
Supplement: Supplementary file 2 — Supplementary Material 2 [file 12903_2025_6342_MOESM2_ESM.docx]

Scopus:

TITLE-ABS-KEY(Early Childhood Oral Health Impact Scale OR ECOHIS) AND ( LIMIT-TO ( EXACTKEYWORD,"Human" ) ) AND ( LIMIT-TO ( LANGUAGE,"English" ) )

Pubmed:

Early Childhood Oral Health Impact Scale[Title/Abstract] OR ECOHIS[Title/Abstract]

EMBASE:

('early childhood oral health impact scale OR ECOHIS') AND [english]/lim

CINAHL and Dentistry and Oral Science Source:

Early Childhood Oral Health Impact Scale OR ECOHIS

Web of science:

((TI=(Child OR Children)) OR AB=(Child OR Children)) OR AK=(Child OR Children) AND ((TI=(early childhood oral health impact scale OR ECOHIS)) OR AB=(early childhood oral health impact scale OR ECOHIS)) OR AK=(early childhood oral health impact scale OR ECOHIS)
